# Spatial genetic structure of the invasive tree *Robinia pseudoacacia* to determine migration patterns to inform best practices for riparian restoration

**DOI:** 10.1093/aobpla/plaa043

**Published:** 2020-08-24

**Authors:** Sakiko Yaegashi, Tatsuo Omura, Kozo Watanabe

**Affiliations:** 1 Department of Civil and Environmental Engineering, University of Yamanashi, Kofu, Yamanashi Prefecture, Japan; 2 New Industry Creation Hatchery Center (NIChe), Tohoku University, Aoba-yama, Sendai, Japan; 3 Center for Marine Environmental Studies (CMES), Ehime University, Bunkyo-cho, Matsuyama, Ehime, Japan; 4 Department of Civil and Environmental Engineering, Ehime University, Matsuyama, Ehime, Japan

**Keywords:** Elimination, false acacia, gene flow, invasive species, river management

## Abstract

The black locust *Robinia pseudoacacia* (Robinieae, Fabaceae) is a common invasive riparian tree in Japan. There are less effective management strategies to remove the tree from the riparian area because of its quickly established high population. We investigated the expansion patterns of *R. pseudoacacia* through sympatric (i.e. between high- and low-water channel (HWC/LWC) within a study site) and allopatric (i.e. along river corridor) dispersal in the Tama River (Tokyo, Japan). Four microsatellites were used to examine the effects of gene flow on six populations in three sites. These subpopulations showed small genetic distance (i.e. no barrier or slightly limited) and genetically mixed population structure. It indicated that both sympatric and allopatric dispersals were active. Many migrants were younger individuals (i.e. <5 years old) and were found in the LWC area. Thus, the LWC could receive more migrants than the HWC through both types of dispersals. In addition, our age and genetic structure analyses reveal that recruited individuals likely settled immediately after the clearing project of *R. pseudoacacia* through sympatric dispersal. It appears that the migration by allopatric dispersal occurred following this. For the effective management of *R. pseudoacacia*, migrants should be removed regularly following initial removal of invaders during site restoration.

## Teaser

The black locust *Robinia pseudoacacia* is a common invasive species inhabiting Japanese river banks. It is problematic both for ecosystem and disaster prevention. We investigated the expansion strategy of the tree based on gene flow on three sites, in Tama River using microsatellites. A clearing project was conducted on a site. Migrants seemed to be settled immediately on the riverside after the clearing project from the close population first, and then river water could carry new migrants. For the plant’s effective management, migrants should be removed regularly following the initial removal of invaders during site restoration.

## Introduction

A fundamental challenge in conservation biology is to gain insights into the ecological process of invasions, such as the patterns of expansion and immigration strategies of alien species (Sakai *et al.* 2001; Washitani 2001). The black locust (*Robinia pseudoacacia*, Robinieae, Fabaceae) is a common invasive tree in riparian areas in Japan (Miyawaki and Washitani 2004). The black locust is native to the southern Appalachian Mountains in North America (Keresztes 1980) but was introduced to the world for forestation and apiculture purposes (Keresztes 1980; Cierjacks *et al.* 2013; Vítková et al. 2017). Its first introduction to Japan was reported in 1873 (Sakio 2009), and it has spread quickly throughout riparian areas (Maekawa and Nakagoshi 1997). According to a recent National Census on River Environments, *R. pseudoacacia* is now present in 84 % of river basins in Japan (i.e. 97/115 basins) (Ministry of Land, Infrastructure, Transport and Tourism (MLIT), accessed in 2014).

**Figure 1.**
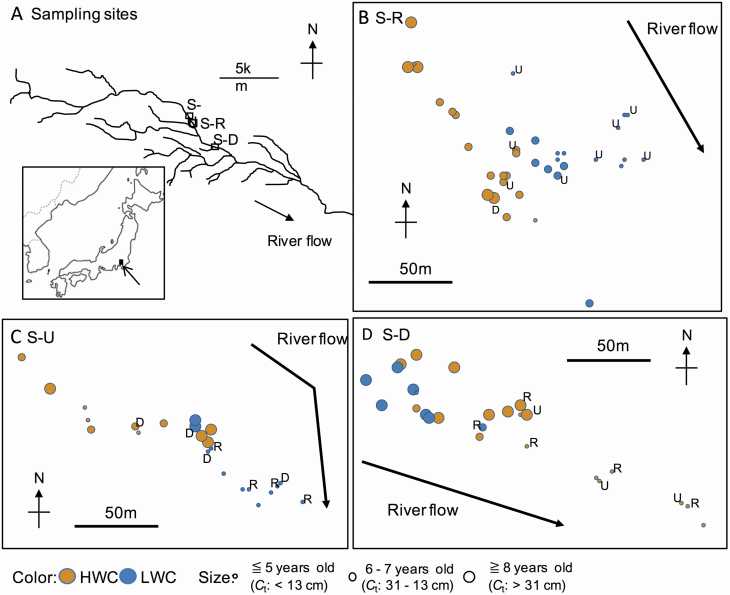
Map of the study area indicating study sites (A: all sampling sites, B: S-R, C: S-U, D: S-D). Each circle indicated the sampling location of each individual. Circle colour distinguishes subpopulation (i.e. HWC or LWC) and size stated the age of individuals. Capital letters indicated individuals assigned to other population. U = assigned to S-U, R = assigned to S-R, D = assigned to S-D.

The expansion of *R. pseudoacacia* has led to two primary problems in riparian areas. The first is the loss of native plant diversity due to invasion. For example, this has greatly affected the chrysanthemum (*Aster kantoensis*) in the Tama River in Tokyo (Washitani *et al.* 1997, reviewed in Cierjacks *et al.* 2013; Vítková *et al.* 2017). The second problem is the reduction of the flood-flow capacity within river channels. When flood events occur, *R. pseudoacacia* tends to be washed out, and its trunks prevent downstream water flow, resulting in high water levels during floods (Asaeda *et al.* 2010, 2011). Therefore, because of these issues related to ecological conservation and flood control, in 2002, the MLIT tried to remove trees of *R. psudoacacia* in the area of S-R (Fig. 1) and the surrounding soils including their roots from the riparian area in the Tama River (Ogawa *et al.* 2011). However, the *R. psudoacacia* population recovered after clearing.

**Figure 2.**
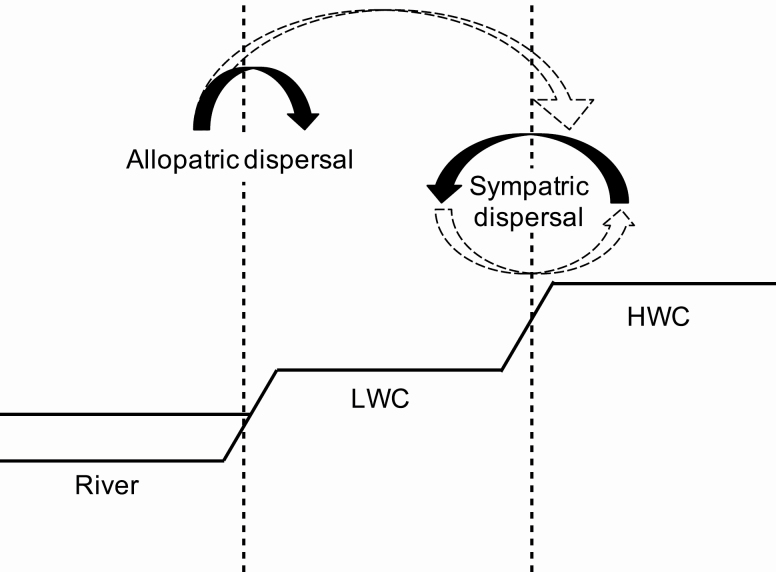
The image of migration flow in riparian area. Arrows indicated how migrants come.

Understanding the means for expansion can tell us about more effective methods to clear it from the riparian area. Propagation of *R. pseudoacacia* varies across the different phases of its life cycle, such as seeds, underground stems, basal shoots, epicormic branches and damaged trunks. Several previous studies reported that allopatric dispersal (i.e. migrants carried by river water along riverine corridors; Fig. 2) occurred primarily via seed dispersal (Säumel and Kowarik 2013), whereas robust sympatric dispersal (i.e. reproduction occurred within small area; Fig. 2) was reported to be because of the extension of underground stems (Jung *et al.* 2009; Kurokochi *et al.* 2010; Asaeda *et al.* 2011). Seed dispersal occurs primarily in nearby areas because of gravity and wind (Robinson and Handel 1993; Morimoto *et al.* 2010). Sprout outbreaks and the extension of underground stems have been studied using GIS and field digging observations (Jung *et al.* 2009, reviewed in Cierjacks *et al.* 2013). Several genetic analyses revealed the distribution of clones (Chang *et al.* 1998; Lian *et al.* 2009) and strong gene flow in basins (Lian *et al.* 2009; Kurokochi *et al.* 2010). Seeds showed inbreeding depression (Cierjacks *et al.* 2013; Yuan *et al.* 2013).

We predicted that allopatric dispersal from outside populations was more active in low-water channel (LWC), which is at lower elevation, near river channel and frequently inundated (Fig. 2), than in high-water channel (HWC), which is at a considerably higher elevation and less inundated (Fig. 2). In contrast, sympatric dispersal in a native population was more common in HWC. Previous studies reported that the HWC showed higher densities of *R. pseudacacia*’s re-shootings than did the LWC (Asaeda *et al.* 2010, 2011; Asaeda and Rashid 2014). Sapling density was reduced at middle elevations (i.e. 2–3 m) but was higher at low (i.e. 0–2 m) and higher elevations (i.e. >3 m) (Asaeda *et al.* 2011). Although these phenomena suggest that the LWC, compared with HWC, has a different expansion process, this has not been tested genetically.

In this study, we examined the spatial genetic structure of *R. pseudoacacia* populations to infer its spatial expansion patterns focused on allopatric and sympatric dispersal. Our main objectives were: (i) to investigate the existence of two patterns of dispersal (i.e. sympatric and allopatric dispersal) by its gene flow, (ii) to reveal how these dispersals were related to population establishment in HWC and LWC area and (iii) to estimate the process of population establishment after the restoration project. To approach these objectives, we examined spatial genetic variation using highly polymorphic microsatellites for *R. pseudoacacia* sampled from three study sites, including a restoration site. Because microsatellites are able to reveal the genetic structure and dispersal pattern at a narrow geographic scale (i.e. within a basin, plant community) (Lian *et al.* 2009; Kurokochi *et al.* 2010, and Jung *et al.* 2009; Love *et al.* 2013; Hevroy *et al.* 2018), we employed the microsatellites to determine migration pattern of the invasive locust tree *R. pseudoacacia* in Tama River (Tokyo, Japan) to consider efficient riparian restoration.

## Methods

### Study sites and sampling

Field surveys were conducted in two subpopulations (LWC and HWC; Fig. 2) at three sites (six subpopulations in total) along the Tama River (Tokyo, Japan). The three sites are located at 53.5, 52.0 and 41.5 km (named site upstream (S-U), site restoration (S-R), site downstream (S-D), respectively; Fig. 1) from the river mouth. A restoration project had been conducted at the S-R km in 2002. Trees including *R. pseudoacacia* were removed from the LWC at the S-R to restore gravel bars. Simultaneously, surface soil, including tree roots, was also removed to prevent reproduction of *R. pseudoacacia* (Ogawa *et al.* 2011). The other two study sites were located upstream and downstream of the S-R. There is a weir that leads water into the Tamagawa aqueduct upstream of the S-U. The S-D is located downstream of the confluence of the Tama River and the other two tributaries. According to vegetation maps (Ikeya 2001), *R. pseudoacacia* showed rapid expansion in all three sites. That is, at the S-U, the expansion was from 1.2 ha in 1979 to 7.0 ha in 1995, and at the S-R, it was from 1.0 ha in 1979 to 8.55 ha in 1995. In particular, the *R. pseudoacacia* habitat at the S-D in 1995 expanded 12.5 times compared to that in 1979 (i.e. from 0.55 ha in 1979 to 6.85 ha in 1995). Takahashi and Minagawa (2007) reported that the population of *R. pseudoacacia* at S-R increased exponentially (i.e. from 12 individuals in 1982 to 2435 individuals in 1993). According to the reported water levels from January 1989 to December 2009 at 1.5 km downstream for site S-D (Fig. 1) (Water Information System by MLIT, accessed in 2014), the LWC area was inundated for 806 days (40.3 days per year on average). In contrast, the HWC was covered by water only for 3 days (0.15 days per year on average).

Between 28 August and 18 September 2009, we collected sprout leaves from each tree on the right-side shore at S-U and S-R and on the left-side shore at S-D. The range of other sampling sites was adequate to be nearly as large as that in S-R. The leaves were stored at −20 °C until genetic analysis. We also recorded latitude, longitude, locations (i.e. HWC or LWC) and the circumference of the trunk at 120 cm height (*C*_t_) from each tree that we sampled. A previous study reported that *C*_t_ was correlated with the age of each tree (Takahashi and Minagawa 2007). Distance from the riverside of the main stream (*D*_r_) was measured using the Google Earth tool on the basis of the most recent aerial photos of our field sampling.

### DNA extraction and microsatellite genotyping

We genotyped 86 different trees from the three sites (Table 1). Each ~50 mg leaf was frozen in liquid nitrogen and crushed with a pestle. The crushed leaves were incubated at 60 °C for 30 min with 500 µL of cetyltrimethyl ammonium bromide buffer. DNA was isolated with chloroform extraction and isopropanol precipitation. After the extraction, DNA was stored in TE buffer at −20 °C. DNA concentrations were measured using a NanoDrop-1000 spectrophotometer (Thermo Fisher Scientific, Waltham, UK).

**Table 1.** Genetic diversity of *Robinia pseudoacacia* at each site measured at four polymorphic microsatellite loci. *N* = number of individuals genotyped, *D*_r_ = average distance from the riverside, *C*_t_ = average of the 120 cm high circumference of trunk (cm), *N*_a_ = average number of alleles per locus, *N*_e_ = number of effective alleles, *H*_o_ = observed heterozygosity, *H*_e_ = expected heterozygosity and *F*_is_ = fixation index.

Site	Population	*N*	*D*_r_	*C*_t_	*N*_a_	*N*_e_	*H*_o_	*H*_e_	*F*_is_
S-U	HWE	11	35.7	26.0	5.25	3.517	0.386	0.708	0.423
	LWC	12	58.3	9.3	8.25	6.169	0.521	0.849	0.337
	All	23	17.3	47.5	9.25	5.405	0.457	0.808	0.411
S-R	HWE	19	81.5	26.8	9.50	5.590	0.487	0.789	0.337
	LWC	18	83.7	6.1	8.50	4.637	0.583	0.771	0.241
	All	37	82.6	16.8	11.25	5.879	0.534	0.795	0.314
S-D	HWE	18	43.8	28.3	7.75	4.326	0.333	0.728	0.535
	LWC	8	35.9	37.6	4.50	2.674	0.438	0.515	0.028
	All	26	41.4	31.2	8.00	4.080	0.365	0.677	0.452

We genotyped all samples at four microsatellites (Rops02, Rops04, Rops05 and Rops08; Lian and Hogetsu 2002). PCR products showed clear peaks during subsequent fragment analysis, even when our budget was limited. We ran PCR with a Veriti 96-well thermal cycler (Applied Biosystems, Foster City, CA, USA) in 10 μL reaction volumes with 10 ng template DNA, 0.25 U *Taq* DNA polymerase (TaKaRa, Tokyo, Japan), 1× PCR buffer (TaKaRa), 2.5 mM MgCl_2_ and 0.4 mM dNTP (TaKaRa). PCR cycling conditions were 2 min at 95 °C, followed by 35 cycles of 30 s at 95 °C, 30 s at annealing temperature for each primer (Lian and Hogetsu 2002), 1 min at 72 °C and a final extension step of 5 min at 72 °C. We analysed fragment sizes on an ABI 310 automated sequencer (Applied Biosystems) with GeneScan (Applied Biosystems). We estimated fragment sizes with a GeneScan 500 ROX size standard (Applied Biosystems). Although we employed fewer microsatellites than standard microsatellite studies, these markers reliably amplified our DNA. As several previous studies that focused on the gene flow of trees also employed four microsatellite loci (Marzouki *et al.* 2009; Nielsen and Kjær 2010; Pandey and Geburek 2010, 2011), studying the evaluation of gene flow is possible.

### Data analyses

We calculated five genetic indices: the number of alleles (*N*_a_), the number of effective alleles (*N*_e_), observed heterozygosity (*H*_o_), expected heterozygosity (*H*_e_) and the fixation index (*F*_is_) within each population using GenAlEx (version 6.4; Peakall and Smouse 2006). We tested for linkage disequilibrium (LD) and deviation from the Hardy–Weinberg equilibrium (HWE) for each population and each locus using GENEPOP (version 4.2; Raymond and Rousset 1995) with a likelihood test (settings: dememorization, 1000; batches, 100; iteration per batch, 1000). We adjusted the significance levels using the Bonferroni correction. We estimated null allele (ENA) frequencies and global *F*_st_ with and without the ENA correction using FreeNA (Chapuis and Estoup 2007) and the EM algorithm (Dempster *et al.* 1977) with 1000 permutations.

We delineated populations using a model-based Bayesian clustering analysis of all 86 genotyped individuals in STRUCTURE (version 2.3.2; Pritchard *et al.* 2000). The correlated allele frequency model (Falush *et al.* 2003) infers population structure where there are *K* genetic groups (*K* is unknown *a priori*). The model estimates the log likelihood (ln*P*[*K*]) for each *K* (named No. *K*-pop) and an optimal *K* can be chosen on the basis of the highest standardized second-order rate of change (Δ*K*) of ln*P*(*K*) (Evanno *et al.* 2005). For comparing the relationship between subpopulations and those among sites, we also chose two and three as number of *K*-pop. We performed 20 runs of 100 000 iterations with a burn-in of 50 000 for each *K* ranging from 1 to 20 using the admixture model. We used a uniform prior for α, the parameter representing the degree of admixture, with a maximum of 10.0, and set Alphapropsd to 0.05. Lambda, the parameter representing the correlation in the parental allele frequencies, was estimated in a preliminary run using *K* = 1. The prior *Fst* was set to default values (mean ± SE, 0.01 ± 0.05). We employed STRUCTURE HARVESTER (Earl and von Holdt 2011) to calculate Δ*K* and CLUMPP version 1.1.2 (Jakobsson and Rosenberg 2007) to find optimal alignment clusters across multiple runs with following approaches: LargeKGreedy method and the *G*′ pairwise matrix similarity statistics. Each individual was assigned to the genetic population having the highest *q-*value (i.e. assignment proportion to each *K*-pop).

Correlation analyses were conducted among each individual’s geographic distance from the riverside of the main stream, the assignment proportion to each *K*-pop with the most assigned individuals in each site (*QK*) and its trunk circumference (*C*_t_) using the test for association/correlation between paired samples in R (Hollander and Wolfe 1973a; Best and Roberts 1975). In addition, seven measurements (i.e. *N*_e_, *N*_a_, *H*_o_, *H*_e_, *F*_is_, mean geographic distance from the riverside, the mean 120 cm high circumference of the trunk) were compared between HWC and LWC with Wilcoxon rank sum and signed-rank tests in R (Bauer 1972; Hollander and Wolfe 1973b).

A frequency-based assignment test (Paetkau *et al.* 2004) was performed to find putative migrants GenAlEx (version 6.4; Peakall and Smouse 2006). All individuals were divided into three prior populations based on sampling sites. Each individual was assigned to the population with the highest log likelihood.

We calculated pairwise *F*_st_ (Wright 1978) among the six subpopulations or two populations and two subpopulations (i.e. S-U, HWC and LWC in S-R and S-D) using Arlequin (version 3.1; Excoffier *et al.* 2005). The pairwise *Fst* value of 0.14 was used as a criterion for genetic differentiation and as 0.26 for slight differentiation because Hamrick *et al.* (1993) reported that *F*_st_ was lower than ~0.14 in species with wind-promoted (=likely no barrier) gene flow and was ~0.26 in species with animal-promoted gene flow. The analysis of molecular variance (AMOVA) provided estimates of genetic differentiation at three hierarchical spatial levels: (i) among sites, (ii) between HWC and LWC within a site and (iii) among individuals within a subpopulation, with 10 000 permutations.

Individual-based genetic distances (*GD*_i_; Smouse and Peakall 1999) were calculated by GenAlEx (version 6.4; Peakall and Smouse 2006) to compare genetic differentiation among the following categories: individuals from (i) within a site or between sites, (ii) HWC or LWC subpopulations within the same site and (iii) HWC or LWC between other sites (e.g. between HWC in S-U and in S-R) and LWC between other sites. The difference in average values in each category was tested by the Tukey–Kramer test using R v 3.5.3. The correlation between *GD*_i_ and *F*_st_ was tested by Spearman’s rank correlation test using R v 3.5.3.

## Result

All four microsatellite loci were polymorphic (*N*_a_ ranged from 8 to 18; **see**  **Supporting Information—Table S1 and Data S1**). Significant LD was detected between Rops02 and Rops05 at the HWC in S-R, and deviations from HWE were shown for Rops02, Rops05 and Rops04 **[see**  **Supporting Information—Table S1****]**. Null alleles were detected for all loci (see Supporting Information—Tables S1 and S2). However, global *F*_st_ among the six subpopulations with the ENA correction (=0.070, range 0.009–0.129; **see**  **Supporting Information—Table S1**) was nearly identical to the global *F*_st_ without the ENA correction (=0.071, range 0.005–0.123; **see**  **Supporting Information—Table S1**). Therefore, we did not exclude any loci from further analyses.

Mean *H*_o_ among the three subpopulations in LWC was significantly higher than that among subpopulations in HWC (d.f. = 11, *V* = 11, *P* < 0.05). Mean *F*_is_ among HWC subpopulations was significantly higher than that among LWC subpopulations (d.f. = 11, *V* = 69, *P* < 0.05). The trend of the other indices (i.e. *N*_a_, *N*_e_ and *H*_e_) at S-U (i.e. the LWC is higher than the HWC) was different from that at the other reaches (i.e. the LWC is less than the HWC).

**Figure 3.**
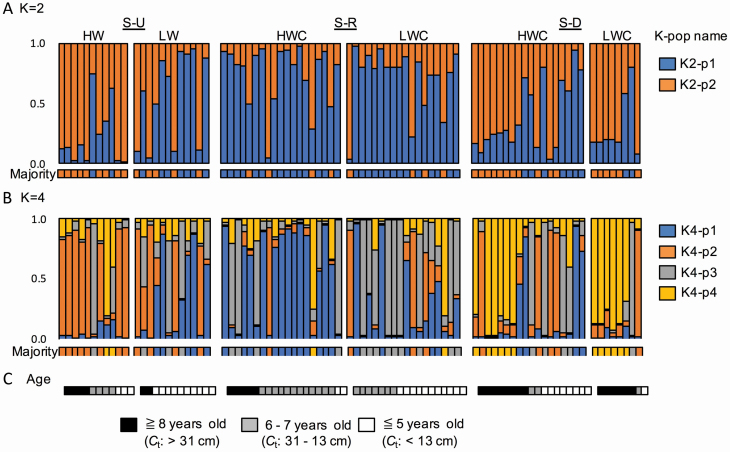
The result of Bayesian clustering analysis (A, B) and the age of each individual (C). Samples are sorted on the basis of whether they come from HWC (left) or LWC (right) areas. Individuals are subsequently sorted by their the 120 cm high circumference of trunk (*C*_t_). Each upper rectangle, containing coloured vertical lines, indicates each individual’s proportion of assignment to each of *K* populations (*K* = 2 and 4, in A, B) based on hierarchical Bayesian clustering analysis. Each lower rectangle indicates the majority of *K*-pop (in A, B).

Bayesian clustering analysis delineated four populations among the 86 genotyped individuals (Fig. 3). The Δ*K* was found in four populations. The dominant *K*-pops were complementary (i.e. *K* = 4; *K*4-p2 in S-U, *K*4-p1 in S-R and *K*4-p4 in S-D). Half of the individuals in the LWC subpopulation in S-R were assigned to *K*4-p3. However, all *K*-pops were found in small numbers at all sites. In the case of *K* = 2, *K*2-p2 was found frequently at HWC in S-U and S-D, whereas *K*2-p1 was found in S-R. Comparing the relationship between *K* = 2 and *K* = 4, *K*2-p1 corresponded to *K*4-p2 and *K*4-p3, whereas *K*2-p1 corresponded to *K*4-p1 and *K*4-p3.

The assignment test showed that the proportion of self-assignment in individuals at HWC was higher than that in individuals at LWC in the S-U and S-R (Table 2). The overall self-assignment probability was 70.9 %. The direction of the assignment was two-directional: from upstream to downstream (e.g. from S-U to S-R) and from downstream to upstream (e.g. from S-D to S-R). Most migrants (i.e. individuals assigned to non-sample sites) were <5 years old and located in the LWC area in S-U and S-R and in the HWC area in S-D (Fig. 1; Table 2). In particular, migrants in S-R were more prevalent in the LWC area.

**Table 2.** The result of the assignment analysis.

			No. of individuals			Proportion of	
			Assigned site				
			S-U	S-R	S-D	Self-assignment	Non-self-assignment
Sampled site	S-U	HWC	10	0	1	0.909	0.091
		LWC	5	4	3	0.417	0.583
	S-R	HWC	2	16	1	0.842	0.158
		LWC	6	12	0	0.667	0.333
	S-D	HWC	3	4	11	0.611	0.389
		LWC	0	1	7	0.875	0.125

All *F*_st_ paths between HWC and LWC within a site did not show any barrier (i.e. <0.14; **see**  **Supporting Information—Table S3**). The HWC in S-D showed strong connectivity in all subpopulations, whereas most links connected to the LWC in S-D meant genetic differentiation. On average, *F*_st_ in HWC, LWC, between HWC and LWC within a site and between sites was 0.098, 0.122, 0.046 and 0.111, respectively. In the case of four populations (two sites and two subpopulations in S-R), all *F*_st_s were <0.12 (i.e. no differentiation among site/subpopulations). The *F*_st_ values related to S-D were higher than those between S-U and S-R.

**Figure 4.**
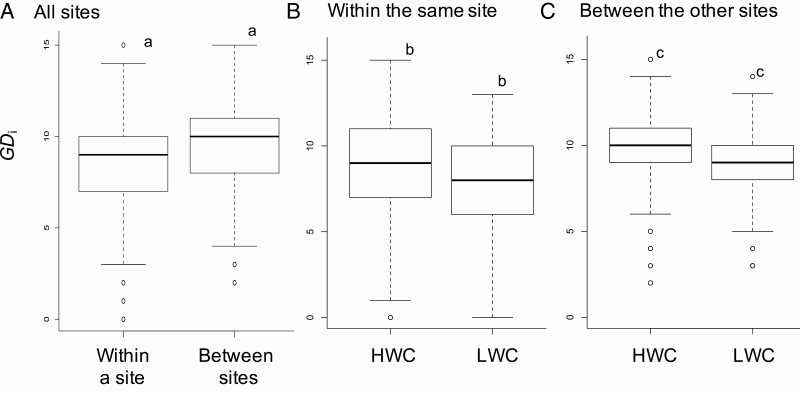
Comparison of individual-based genetic distance (*GD*_i_). (A) All sites, (B) within the same site, (C) between the other sites (e.g. between HWC in S-U and HWC in S-R). HWC = comparison between individuals sampled from HWC; LWC = comparison between individuals sampled from LWC. Bold line is the median, the boxes mean the range of 25–75 % and outsiders were open circle. The small letters indicated that each combination showed *P* < 0.01.

Of note, the average *GD*_i_ within a site was significantly lower than that between sites (*P* < 0.01; Fig. 4). The average *GD*_i_ in the LWC subpopulation within the same site was also significantly lower than that in the HWC subpopulation within the same site (*P* < 0.01; Fig. 4A). In the comparison of *GD*_i_ between sites, the *GD*_i_ at the LWC was significantly lower than that at the HWC (*P* < 0.01; Fig. 4B). In addition, the number at LWC between other sites was also significantly lower than between LWC and between LWC and HWC among other sites (*P* < 0.01; Fig. 4C). On average, *GD*_i_s were 8.84 at HWC within a site, 9.97 between sites, 7.41 at LWC within a site, 9.00 between sites, 8.57 between HWC and LWC within a site and 9.46 between sites. Individuals having the same genotype (i.e. *GD*_i_ = 0) were found within the same HWC subpopulations, within the same LWC subpopulation and among the HWC and LWC subpopulations in the same site. These genotypes were found in the range of 3–42 m. Although the *GD*_i_ was not correlated to *F*_st_ in the case using all subpopulation pairs (*r* = 0.39, *P* > 0.05), it showed significant positive correlation in the case of pairs excluding *F*_st_ related to HWC in S-D (*r* = 0.83, *P* > 0.01). Thus, the pairwise *F*_st_ values related to the HWC appeared to be higher due to the effect of sample size.

The AMOVA detected significant genetic variation at all three hierarchical levels, with 5.19 % of the total variation partitioned to the among-sites level (d.f. = 2, *P* < 0.01), 4.22 % between HWC and LWC subpopulations within a site (d.f. = 3, *P* < 0.01) and 90.59 % among individuals within a subpopulation (d.f. = 166, *P* < 0.01).

The *C*_r_s were correlated to several *Q*_*K*_ (*Q*_*K*2_p2_ in S-U, *r* = 0.43, *P* < 0.05; *Q*_*K*4_p2_ in S-U, *r* = 0.47, *P* < 0.05), and the *D*_r_s were also correlated to several *Q*_*K*_ (*Q*_*K*4_p1_ in S-R, *r* = 0.39, *P* < 0.05). The correlations between *D*_r_ and *C*_t_ were not significant at any of the sites. The mean *C*_t_ in the LWC at S-U (=9.3 cm) and S-R (=6.1 cm) was significantly smaller than in the HWC (S-U = 26.0 cm and S-R = 26.8 cm) (*P* < 0.01), whereas no significant difference was found at S-D (*P* > 0.05). In S-R, migrants were more prevalent at the LWC, whereas the oldest individuals at the LWC were found at the border with HWC. In S-R, the oldest individuals at the LWC were found on the border with HWC.

## Discussion

### Gene flow of *Robinia pseudoacacia* in riparian areas

To address the invasion strategies of black locust *R. pseudoacacia* in the Tama River, we examined its genetic structure and gene flow using four microsatellites. The *R. pseudoacacia* in our study showed both sympatric and allopatric gene flow actively. We initially focused on the allopatric dispersal. The subpopulation-based genetic distance (i.e. pairwise *F*_st_) within a site indicated no barrier, and the individual-based genetic distance (i.e. *GD*_i_) also showed the closest relationship in other *GD*_i_s. The individuals with the same genotype, who could be clones or seeds from the same origin, were found only within the same site. In addition, dominant *K*-pops were found in both subpopulations within a site. A previous genetic study also reported the occurrence of close relatives within ~50 m^2^ (Kurokochi and Hogetsu 2014). These results indicated that active gene flow occurred in a narrow area. This active gene flow could be led by seed and pollen dispersal. The dispersal of *R. pseudoacacia* was conducted by wind in their native habitat (Robinson and Handel 1993). Partial seed dispersal occurred close to the parents by gravity and wind (Morimoto *et al.* 2010; Cierjacks *et al.* 2013). Most pollen could be transported to nearby individuals by animal pollinators (Stacy *et al.* 1996) or by wind (Geng *et al.* 2008; Isagi *et al.* 2007). At our study sites, strong sympatric dispersal was believed to have occurred due to seed and pollen dispersal. However, it is difficult to determine its direction. High-water channel subpopulations could provide seeds to those of LWC as there were older individuals (i.e. >8 years old) at HWC in S-U and S-R. Reverse direction of gene flow led could also have possibly occurred by wind and animal. This needs to be further studied in the future.

The allopatric dispersal was also active. Furthermore, it may have occurred in two directions: from upstream to downstream (e.g. from S-U to S-D) and from downstream to upstream (e.g. from S-D to S-R), although we predicted the gene flow from upstream to downstream conducted by river water. Most pairwise *F*_st_s indicated no barrier, and three of them related to the LWC subpopulation in S-D showed a little genetic differentiation (i.e. higher *F*_st_ than wind-promoted one). All individuals in each *K*-pop were found at all sites, although dominant *K*-pop was complicated. The migrant analysis also supported the exchange of individuals among study sites. The *R. pseudoacacia* was known to disperse at a long distance from forested mountains or upland (Kurokochi *et al*. 2010; Lian *et al.* 2009; Love *et al.* 2013; Säumel and Kowarik 2013; Hevroy *et al.* 2018). Its pollen could disperse several kilometres (Lian *et al.* 2009; Kurokochi *et al.* 2014). Its seed could be carried by animals such as birds and humans (reviewed in Cierjacks *et al.* 2013; Vítková *et al.* 2017). Not only water flow but also these biological factors could contribute to the allopatric gene flow in two directions.

It was possible that the recruitments occurred in a site and migrants coming from other site grew and established their populations easier in the LWC area than in the HWC one. The *GD*_i_s for LWC within a site or between sites were significantly lower than those for HWC. The fixation indices in the LWC showed lower than the HWC. These facts meant that the LWC subpopulations were more homogenized than the HWC subpopulations. In S-U and S-R, most migrants found in the LWC area, and the average age of LWC subpopulation was younger than that in the HWC subpopulation. It supported the fact that the LWC received more recruitments and migrants. In previous studies, the *R. pseudoacacia* required bright conditions for seed establishment (Groninger *et al.* 1997), and tends not to propagate new ramets in areas already occupied by former residents (Jung *et al.* 2009). These mean that open space is needed for their growth. Since *R. pseudoacacia* is vulnerable to flooding related to its height and the length of underground stems (Asaeda *et al.* 2010), the old population can be washed away easily. As a result, a suitable environment to grow new populations had been created. Migrants were also present in the HWC which was likely to carry by past large flood or animals.

### Establishment *R. psudoacacia* population after clearing project

The restored site the S-R provides an opportunity to observe the recovery of *R. pseudoacacia* populations after clearing. The restoration project was conducted ~7 years ago before this study and removed not only tree bodies and also surface soils, including tree roots (Ogawa *et al.* 2011) to prevent population from re-establishing. Therefore, it is likely that the re-established population was derived from migration from outside of restoration area or remaining seed rather than from remaining roots in this area. However, the oldest seven individuals in LWC (named OL) were almost 6–7 years old, corresponding to the year of project completion.

The establishment of the new population at the LWC seemed to be started from the boundary of the HWC. The Bayesian clustering analysis indicated that different *K*-pops (i.e. *K*2-p1, *K*4-p1 and *K*4-p3) with other sites establish their populations in both HWC and LWC area. Most OL individuals also were assigned to these *K*-pops and located in the boundary between HWC and LWC. Moreover, the OL individuals distributed in the boundary between the HWC and the LWC areas. The migrants from HWC subpopulation could contribute to the population establishment at first rather than soil seed bank or remaining root. Next, the migration by allopatric dispersal occurred after the first establishment. Migrants from S-U found in the place close to the river and were quite younger (i.e. <5 years old). They might be carried by river water. The allopatric migration would be delayed than the sympatric one even though migrants came from close areas (i.e. ~1–1.5 km depart from). In conclusion, it is needed for effective clearing of *R. pseudoacacia* in riparian areas to remove not only its tree body but also surface soil. Subsequently, it is necessary to deal with individuals carried by allopatric dispersal.

## Conclusions

We investigated the gene flow and genetic structure of the invasive tree *R. pseudoacacia* using four polymorphic markers in the three riparian sites and six subpopulations of the Tama River based on 86 individuals.

1. Both sympatric and allopatric dispersals were active in our study area. The genetic distance based on subpopulations indicated that no barrier level and the dispersals of pollen and seed would have occurred within a site by wind or animals. The allopatric dispersal also shown to be active (i.e. no barrier or a little limited) and two-directional (i.e. from upstream to downstream, from downstream to upstream). It could be conducted by seed dispersal not only river flow but also animal and pollen dispersal.
2. The LWC area seemed to have the potential to receive migrants from both other sites and the same site rather than the HWC. Most migrants were young (i.e. <5) and distributed at the LWC area in S-R and S-U. The *R. pseudoacacia* needs bright and open environments such as the LWC area where old populations are washed away by frequent floods.
3. After the restoration project, the first migration seemed to occur at the boundary of HWC and LWC by sympatric dispersal. Subsequently, migrants could be carried by river flow.
4. To successfully clear *R. pseudoacacia* from riparian areas, it is necessary first to remove trees from whole area. As allopatric dispersal through the river corridor also provides migrants from outside, migrants should be removed regularly.

## Supporting Information

The following additional information is available in the online version of this article—

**Table S1.** Total *N*_a_ for each locus, null allele frequency and global *F*_st_ with/without the ENA correction.

**Table S2.** Estimates of null allele frequency and loci with deviations from Hardy–Weinberg equilibrium (HWE) by probability test at each population.

**Table S3.** Pairwise *F*_st_ and average *GD*_i_ between subpopulation/site (above the diagonal) and *P*-value (below the diagonal).

**Data S1.** Sampling site, sample name, subpopulation (high-water channel (HWC) or low-water channel (LWC)), location, each locus genotyping data *C*_t_ (average of the 120 cm high circumference of trunk (cm)) and *D*_r_ (distance from the riverside of the main stream).

plaa043_suppl_Supplementary_Tables

plaa043_suppl_Supplementary_Data_S1
