# Supplementary material for: Spatial genetic structure of the invasive tree Robinia pseudoacacia to determine migration patterns to inform best practices for riparian restoration
Source: AoB Plants. 2020 Aug 24;12(5):plaa043. doi: 10.1093/aobpla/plaa043 (PMC7586742; doi:10.1093/aobpla/plaa043)
Supplement: plaa043_suppl_Supplementary_Tables [file plaa043_suppl_supplementary_tables.docx]

Supporting Information S1. Total *N*_a_ for each locus, null allele frequency, and global *F*_st_ with/without the ENA correction.

|  |  | Null allele frequency | | Global *F*_st_ | |
| --- | --- | --- | --- | --- | --- |
| Locus | Na | Average | Maximum | Without ENA | With ENA |
| Rops02 | 18 | 0.144 | 0.245 | 0.005 | 0.009 |
| Rops04 | 15 | 0.266 | 0.350 | 0.111 | 0.101 |
| Rops05 | 14 | 0.145 | 0.253 | 0.123 | 0.129 |
| Rops08 | 8 | 0.051 | 0.227 | 0.037 | 0.039 |

Supporting Information S2. Estimates of null allele frequency and loci with deviations from HWE by probability test at each population. H = HWC, L = LWC.

| Site |  | Rops02 | Rops04 | Rops05 | Rops08 | Average | HWE |
| --- | --- | --- | --- | --- | --- | --- | --- |
| S-U | H | 0.158 | 0.350 | 0.171 | 0.000 | 0.170 | Rops02, Rops04, Rops05 |
|  | L | 0.116 | 0.320 | 0.122 | 0.000 | 0.140 | Rops02, Rops04, Rops05 |
| S-U | H | 0.245 | 0.136 | 0.168 | 0.082 | 0.158 | Rops02 |
|  | L | 0.151 | 0.273 | 0.253 | 0.000 | 0.169 | Rops04, Rops05 |
| S-D | H | 0.192 | 0.328 | 0.152 | 0.227 | 0.225 | Rops02, Rops04 |
|  | L | 0.000 | 0.187 | 0.000 | 0.000 | 0.047 | non |
|  |  |  |  |  |  |  |  |

Supporting Information S3 Pairwise *F*_st_ and average *GD*_i_ between subpopulation/site (above the diagonal) and *p*-value (below the diagonal). NS: non-significance, *: *p* < 0.05. **: *p* < 0.01.

a) *F*_st_ for All subpopulations

|  |  | S-U | |  | S-U | |  | S-D | |  |
| --- | --- | --- | --- | --- | --- | --- | --- | --- | --- | --- |
|  |  | HWC | LWC |  | HWC | LWC |  | HWC | LWC |  |
| S-U | HWC | - | * |  | ** | ** |  | ** | ** |  |
|  | LWC | 0.062 | - |  | NS | NS |  | NS | ** |  |
| S-R | HWC | 0.130 | 0.013 |  | - | * |  | ** | ** |  |
|  | LWC | 0.126 | 0.016 |  | 0.033 | - |  | ** | ** |  |
| S-D | HWC | 0.114 | 0.039 |  | 0.051 | 0.084 |  | - | NS |  |
|  | LWC | 0.249 | 0.138 |  | 0.154 | 0.213 |  | 0.044 | - |  |

b) *F*_st_ for 2 sites and 2 subpopulations in S-R

|  |  | S-U | S-R |  | S-D |
| --- | --- | --- | --- | --- | --- |
|  |  |  | HWC | LWC |  |
| S-U |  | - | NS | ** | ** |
| S-R | HWC | 0.026 | - | ** | ** |
|  | LWC | 0.056 | 0.050 | - | ** |
| S-D |  | 0.080 | 0.115 | 0.088 | - |

| c) *GD*_i_ for All subpopulations |  | S-U |  | S-R |  | S-D |  |
| --- | --- | --- | --- | --- | --- | --- | --- |
|  |  | HWC | LWC | HWC | LWC | HWC | LWC |
| S-U | HWC | 8.491 |  |  |  |  |  |
|  | LWC | 9.659 | 9.652 |  |  |  |  |
| S-R | HWC | 10.306 | 9.241 | 8.865 |  |  |  |
|  | LWC | 9.692 | 8.745 | 8.629 | 7.758 |  |  |
| S-D | HWC | 10.091 | 9.685 | 9.509 | 9.426 | 9.170 |  |
|  | LWC | 9.841 | 8.969 | 8.882 | 9.278 | 7.410 | 4.821 |

d) *GD*_i_ for 2 sites and 2 subpopulations in S-R

|  |  | S-U | S-R |  | S-D |
| --- | --- | --- | --- | --- | --- |
|  |  |  | HWC | LWC |  |
| S-U |  | 9.403 | 9.751 | 9.198 | 9.727 |
| S-R | HWC | 9.751 | 8.865 | 8.629 | 9.316 |
|  | LWC | 9.198 | 8.629 | 7.758 | 9.380 |
| S-D |  | 9.727 | 9.316 | 9.380 | 8.015 |
